# Reduced and delayed myelination and volume of corpus callosum in an animal model of Fetal Alcohol Spectrum Disorders partially benefit from voluntary exercise

**DOI:** 10.1038/s41598-022-14752-3

**Published:** 2022-06-23

**Authors:** Katrina A. Milbocker, Gillian L. LeBlanc, Eric K. Brengel, Khan S. Hekmatyar, Praveen Kulkarni, Craig F. Ferris, Anna Y. Klintsova

**Affiliations:** 1 Department of Psychological and Brain Sciences, University of Delaware, 105 The Green, Newark, DE 19716 USA; 2 Center for Biomedical and Brain Imaging, University of Delaware, Newark, DE 19716 USA; 3 Department of Psychology, Northeastern University, Boston, MA 02115 USA

**Keywords:** Neuroscience, Developmental disorders

## Abstract

1 in 20 live births in the United States is affected by prenatal alcohol exposure annually, creating a major public health crisis. The teratogenic impact of alcohol on physical growth, neurodevelopment, and behavior is extensive, together resulting in clinical disorders which fall under the umbrella term of Fetal Alcohol Spectrum Disorders (FASD). FASD-related impairments to executive function and perceptual learning are prevalent among affected youth and are linked to disruptions to corpus callosum growth and myelination in adolescence. Targeted interventions that support neurodevelopment in FASD-affected youth are nonexistent. We evaluated the capacity of an adolescent exercise intervention, a stimulator of myelinogenesis, to upregulate corpus callosum myelination in a rat model of FASD (third trimester-equivalent alcohol exposure). This study employs in vivo diffusion tensor imaging (DTI) scanning to investigate the effects of: (1) neonatal alcohol exposure and (2) an adolescent exercise intervention on corpus callosum myelination in a rodent model of FASD. DTI scans were acquired twice longitudinally (pre- and post-intervention) in male and female rats using a 9.4 Tesla Bruker Biospec scanner to assess alterations to corpus callosum myelination noninvasively. Fractional anisotropy values as well as radial/axial diffusivity values were compared within-animal in a longitudinal study design. Analyses using mixed repeated measures ANOVA’s confirm that neonatal alcohol exposure in a rodent model of FASD delays the trajectory of corpus callosum growth and myelination across adolescence, with a heightened vulnerability in the male brain. Alterations to corpus callosum volume are correlated with reductions to forebrain volume which mediates an indirect relationship between body weight gain and corpus callosum growth. While we did not observe any significant effects of voluntary aerobic exercise on corpus callosum myelination immediately after completion of the 12-day intervention, we did observe a beneficial effect of exercise intervention on corpus callosum volume growth in all rats. In line with clinical findings, we have shown that prenatal alcohol exposure leads to hypomyelination of the corpus callosum in adolescence and that the severity of damage is sexually dimorphic. Further, exercise intervention improves corpus callosum growth in alcohol-exposed and control rats in adolescence.

## Introduction

Corpus callosum (CC) myelination supports efficient neural signaling between brain hemispheres as well as subcortical to cortical subregions. Myelination of this tract is fundamental for supporting brain growth and circuit refinement during childhood and adolescence. Oligodendrocyte precursor cell (OPC) proliferation and differentiation lead to the induction of myelination in the mammalian brain and first intensifies during the brain growth spurt (BGS), a period of rapid brain growth during the third trimester of pregnancy^1–3^ during which axon length increases dramatically in a process known as fiber scaling^4^. During early childhood (first two years of life), axonal myelination of specific brain circuits is directed by putative circuit functions, some of which include nursing, vision, and sound localization^5^. The rate of myelin development in the brain varies by age and tract as some white matter tracts mature earlier than others^6^. The brain reaches adult size by the end of early childhood (around six years of age), yet regions continue to mature as the ratio of gray to white matter volumes is altered by axonal pruning for homeostatic circuit refinement in late childhood and early adolescence^6–8^. OPCs have been implicated in synaptic pruning coordination in gray matter regions^9–11^. Following synaptic pruning of redundant synapses, the remaining neural circuits become more heavily myelinated to tune the physiological dynamics of salient connections. To achieve this, the organization and thickness of the myelin sheath may be highly variable along a single axon. Functionally-relevant adaptations to the myelin sheath occur frequently in the adolescent and adult brain to optimize signal transduction^12,13^. As a result, early-life and adolescent experiences play a key role in determining the trajectory of tract myelination during circuit refinement. Tomlinson and colleagues^14^ describe several known effectors of postnatal myelination and their neurobiological substrates. Aerobic exercise is a positive effector of myelination as it stimulates OPC proliferation and myelin basic protein (MBP) production which leads to thickening of the myelin sheath. Contrarily, teratogenic exposure or early-life adversity are characterized as negative effectors of myelination as they prevent the differentiation of OPCs and, in extreme cases, cause apoptosis of myelinating oligodendrocytes (reviewed in: Milbocker et al.^15^). Failure to properly refine neural circuitry and optimize neuronal communication leads to the emergence of maladaptive behaviors^16–19^  and disrupts memory consolidation^20.^

A wealth of evidence provided by preclinical and clinical studies indicates that prenatal alcohol exposure (AE) delays the development and myelination of white matter tracts in the adolescent brain. Prenatal alcohol exposure may result in Fetal Alcohol Spectrum Disorders (FASD), a group of related developmental disorders with a high prevalence around the globe, affecting up to 1 in 20 live births in the U.S. annually^21,22^. Specific diagnoses within the FASD continuum vary depending on the timing and severity of AE, which give rise to differential symptomologies and physical dysmorphologies^23^. A heavy early-term exposure often results in distinct facial dysmorphologies, delayed growth, and severe cognitive delays in a readily recognizable case of Fetal Alcohol Syndrome. Late-term exposures, however, give rise to executive function and learning deficits without hallmark physical dysmorphologies due to their overlap with the brain growth spurt, and are thus more difficult to diagnose. Misdiagnosis with other developmental disorders such as Autism Spectrum Disorder or Attention Deficit Hyperactivity Disorder is common. Therefore, late-term exposure FASDs require the special attention of researchers.

While several behavioral and pharmaceutical therapies exist to support infant and child neurodevelopment and help to resolve some behavioral issues resulting from prenatal AE, fewer therapies have been developed to support FASD-affected youth. To create effective, targeted interventions, it is imperative to identify the underlying neuropathology associated with observed disruptions to behavior. It is well-documented that FASD-affected adolescents exhibit reductions to the growth and myelination of the corpus callosum and this structural anomaly is correlated with impaired perceptual reasoning and executive function^24–27^. Furthermore, evidence from preclinical studies using animal models of FASD demonstrates that alcohol teratogenicity reduces corpus callosum volume with the highest impact in males^28^, and prevents OPC proliferation, induces precocial differentiation of OPCs to mature oligodendrocytes, and potentiates the apoptosis of myelinating oligodendrocytes during infancy and adolescence. Moreover, early-life adversity, in the form of stress or teratogenic exposure, has long-term consequences on glia proliferation and function, ultimately impacting the onset and progression of glia-driven processes such as synaptic pruning and myelination in adolescence^29,30^. Indeed, lasting reductions to myelin basic protein production are observed in the corpus callosum of adult rats in models of FASD, suggesting that the function of the circuits involved in learning, memory, and emotion-processing is diminished across the lifespan^31,32^.

To our knowledge, few if any studies have investigated the potential beneficial effect of a behavioral intervention on supporting adolescent myelination in the AE corpus callosum. Behavioral interventions are effective neuroplastic inducers and are more accessible and affordable to implement when compared to pharmaceutical therapies. Previous work has demonstrated that voluntary exercise in adolescence or adulthood restores neuroplasticity in rodent models of FASD^33–38^. Alterations to cerebral tissue growth, connectivity, and physiology are supported by several neurobiological mechanisms which are stimulated by increased aerobic activity. These include increased cerebral perfusion and metabolism via angiogenesis, upregulations to growth factor production and anti-apoptotics (i.e. antioxidants), as well as alterations to neurotransmitter and hormone signaling (reviewed in: Klintsova et al.^35^). As a result, deficits to executive function, anxiety and depressive-like behaviors, coordination and balance, fine motor skills, and spatial memory are partially mitigated^35^. Thus, the benefits of aerobic exercise on brain plasticity, neurogenesis and synaptic remodeling are well-established, yet evidence supporting changes to white matter remain under-investigated^39^. The few studies investigating the impact of aerobic exercise on white matter structure have revealed that exercise has a positive impact on the maturation of multiple white matter tracts, as evidenced by increases in tract volume, myelin sheath thickness and stimulation of oligoglia lineage cell proliferation and differentiation^14,40,41^. Importantly, these structural changes benefit executive function capacity and motor learning in typically-developing animals^41–43^.

Diffusion tensor imaging (DTI) scanning is a powerful tool that allows for noninvasive longitudinal assessment of structural alterations in myelination. Specifically, DTI scanning measures differences in water movement, or diffusivity, in various biological tissues. The direction of water movement through different tissues is quantified into its 3-dimensional vector components—primary (λ_1_), secondary (λ_2_), and tertiary (λ_3_)—and these values are used to calculate fractional anisotropy (FA), axial diffusivity (AD), and radial diffusivity (RD). FA is calculated on a scale from 0 to 1 and describes the degree to which water molecule movement is restricted by surrounding tissue cytoarchitecture. As expected, FA is lowest when measured in fluid-filled ventricles and highest within tightly-packed fiber bundles of the neural white matter. Therefore, it is not surprising that in the typically-developing brain FA values increase across neurodevelopment^44,45^. AD and RD values describe differences in the directionality of water diffusivity which can be interpreted as alterations to tissue microstructure. AD is the primary direction of water movement and tracks the movement of water molecules along fiber bundles in white matter tracts. Changes to AD (λ_1_) occur in cases of altered axonal integrity (i.e. during early development or in cases of axon degeneration). RD represents the average water movement that is perpendicular to the fiber bundles ((λ_2_ + λ_3_)/2). There is an inverse correlation between RD and myelination: RD values decrease across adolescence in the typically-developing brain as water diffusivity is restricted by de novo myelination and thickening of myelin sheaths^44^. FA values represent the foil of the sum of axial and radial diffusivity.

To investigate the effect of voluntary exercise on myelination in a rodent model of FASD, we conducted a longitudinal experiment using noninvasive neuroimaging pre- and post-intervention exposure. Combining the knowledge of developmental myelination with our understanding of FASD-associated neuropathology, we hypothesized that exposure to a positive effector of myelination targeting the peak of adolescent myelination might mitigate the impact of alcohol exposure during the BGS on the trajectory of corpus callosum growth and myelination in adolescence. To test our hypothesis, we have collected two sets of DTI scans (pre- and post- exercise intervention, within-subject) in AE and control rats with and without intervention exposure to investigate changes to myelination by comparing values of FA, AD, and RD in corpus callosum. Importantly, sex was included as a biological factor in this study. This is particularly significant as we are the first to investigate this in the female FASD brain and evidence indicates that the typical progression of myelination across the lifespan is sexually-dimorphic^46,47^.

## Methods

### Rat model of Fetal Alcohol Spectrum Disorders

All rats were acquired, cared for, and methodology was in accordance with the guidelines provided by the University of Delaware’s Institutional Animal Care and Use Committee, the NIH Guide for the Care and Use of Laboratory Animals, and comply with the ARRIVE guidelines (https://arriveguidelines.org), and were approved by the University of Delaware’s Institutional Animal Care and Use Committee (animal use protocol #1134).

 Ten timed-pregnant Long-Evans dams were obtained from Charles River Laboratories (Boston, MA) and litters were culled to ten pups each three days after birth to counterbalance for sex and postnatal treatment group. On the same day, rat pup paws were tattooed using India Ink for identification of each pup in the litter across the duration of the experiment. To investigate the teratogenic impact of binge-like AE during the brain growth spurt on adolescent neurodevelopment, half of each litter was exposed to alcohol during the first two postnatal weeks of life as described in greater detail in Milbocker and Klintsova^37^ and Gursky and Klintsova^48^. A 2020 report discovered that 4% of pregnant women across the United States participate in binge drinking (4 or more drinks during one occasion)^49^. Moreover, England et al.^50^ analyzed national survey data on drug use and health from 2015 to 2018 and found that 1.4% of pregnant women reported binge drinking during the third trimester of pregnancy, highlighting the relevance of studying late-term alcohol exposure using the established rat model of FASD described below^50^.

Briefly, on postnatal days (PD) four through nine, AE pups received a 5.25 g/kg/day (11.9% v/v, divided into two administrations two hours apart) of alcohol in milk substitute via intragastric intubation. An additional milk-only intubation was given to AE pups two hours after the second alcohol dose to compensate for reduced suckling during intoxication. The remaining half of each litter, procedural control pups, was sham-intubated (SI) without liquid administration during the same postnatal period. A 60 μL blood sample was collected from the tail vein of each pup 90 min after the second dose on the first day of treatment (PD four) to measure blood alcohol concentration (BAC)^51^. Blood samples collected from AE rats were analyzed using an Analox GL5 Alcohol Analyzer (Analox Instruments, Boston, MA) and resulted in an average daily BAC of 321 ± 22.8 mg/dL, indicating high AE during the postnatal treatment period. Measuring peak BAC during specific periods of development not only confirms receipt of alcohol, but is used as a significant indicator of brain damage^1,51,52^. The moderate to high BACs resulting from our exposure procedure model the BAC of a pregnant woman during a one-occasion binge-like event, and thus, increase translatability. Patten et al.^53^ provide a complete review of animal models of FASD and their relation to the clinical setting^53^. All animals were weighed daily during the treatment period. Following the last dose on PD nine, all pups were earpunched according to litter number for increased ease of identification during the remainder of the longitudinal study. On PD23, all rats were weaned into same-sex cages of 2–3.

### Voluntary adolescent exercise intervention

On PD30, adolescent rats were randomly re-housed into either modified wheel running cages (WR) or into socially-housed (SH) cages as sedentary controls until PD42–45. Previous studies from our lab and others have demonstrated that a 12-day duration of voluntary exercise is optimal to produce significant structural alterations in the brain and that WR in adolescence and adulthood results in neuroplastic remodeling in the cortex, hippocampus, and cerebellum in rat models of FASD^42,54^. Social housing was maintained for all rats during the entirety of the study to prevent the confounding effects of social isolation on oligodendrocyte maturation and myelination^55,56^. The number of wheel rotations per cage was recorded daily at 09:00 to compare the total running distance between cages containing AE and SI rats of each sex. The circumference of the wheels was used to convert the number of rotations to kilometers run. All rats were weighed at the beginning, middle, and end of the intervention period in order to assess correlations between weight and brain volume. Based on a priori power analyses, a total of 100 pups from 10 litters were generated for this study resulting in an expected sample size of 12 animals per sex/postnatal treatment group/intervention group (8 total groups: F/SI/SH, F/AE/SH, M/SI/SH, M/AE/SH, F/SI/WR, F/AE/WR, M/SI/WR, M/AE/WR).

### Longitudinal magnetic resonance imaging (MRI) scanning

**Figure 1:**
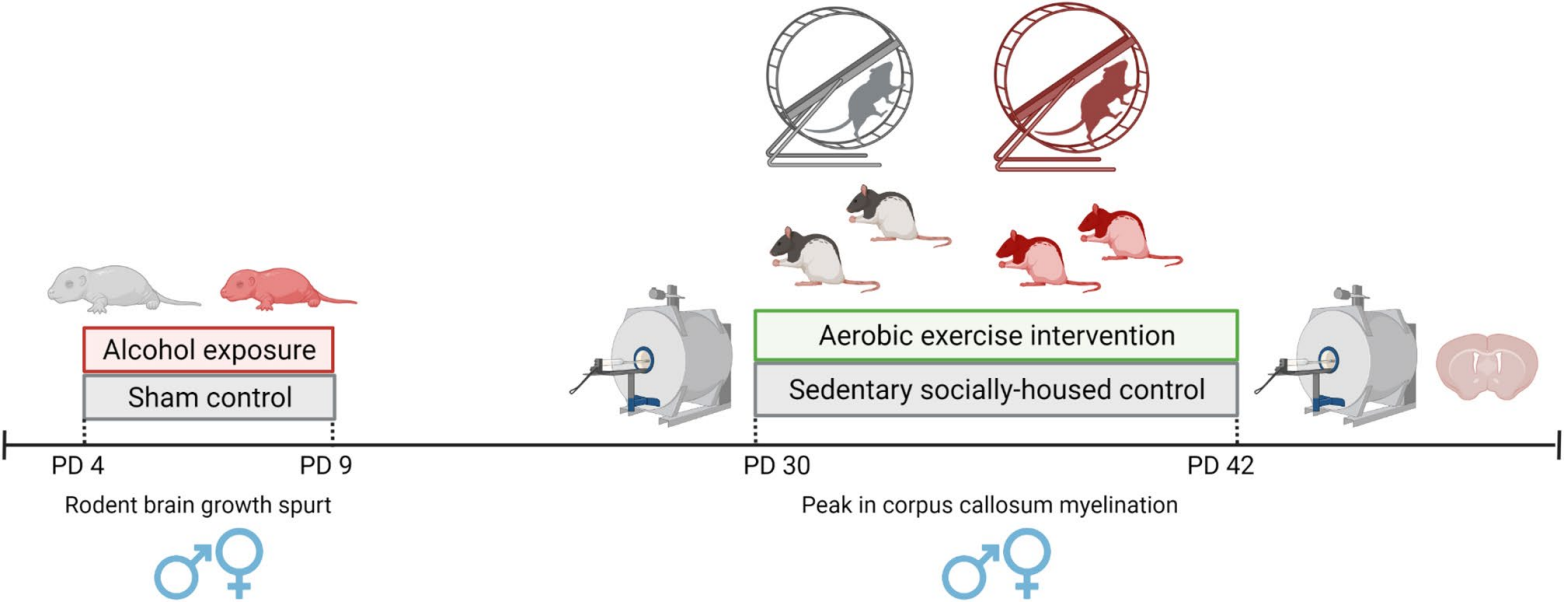
Experimental timeline depicting postnatal treatment (AE/SI) and intervention exposures (WR/SH). Scans were collected twice from each rat pre- and post-intervention. [*Graphic representation of the timeline is created with BioRender.com*].

A novel in vivo neuroimaging protocol was developed to scan the rats once they reached adolescence, pre- and post-intervention in a within-subjects longitudinal study design. Pre-intervention scans were collected from PD27–30 while post-intervention scans were collected from PD42–45 as was feasible given scanner availability. Brain tissue was fixed and collected from all rats between PD45–47 following the final scanning session. An experimental timeline is depicted in Fig. 1.

**Figure 2:**
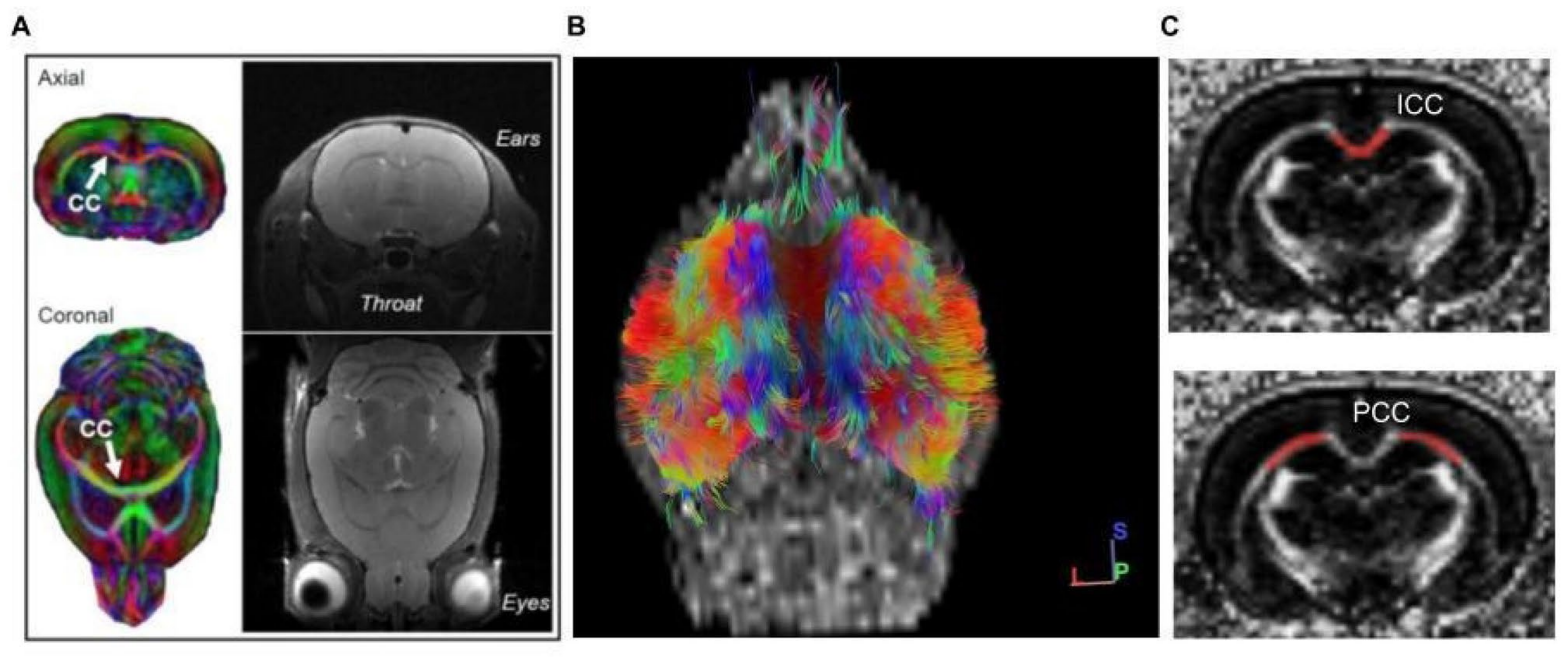
(**A**) Representative images from diffusion tensor imaging (left) and T2-weighted anatomical (right) scanning from an anesthetized male rat on PD30. Scans were analyzed in axial orientation for this study and an arrow indicates the location of the CC in the DTI scans. Reconstruction of DTI images yields data of water diffusivity within 3D tracts as depicted in (**B**, ventral view). Red-blue-green color schema represents fiber orientation with red indicating fibers traveling right to left, blue indicating fibers traveling dorsal to ventral, and green indicating fibers traveling in the rostrocaudal direction. (**C**) Regions of interest (interhemispheric commissural fibers, ICC and cortically-projecting fibers, PCC) contoured in red on grayscale diffusion tensor imaging scans to collect fractional anisotropy, axial and radial diffusivity data.

A series of multimodal scans was captured using a 9.4 Tesla Bruker BioSpec scanner (Bruker; Bellirica, MA) with Paravision 6.2 housed in the Center for Biomedical and Brain Imaging at the University of Delaware to examine structural alterations to corpus callosum across adolescence. A 4-channel rat brain surface array coil was used to collect the images. First, a high resolution 2D T2 weighted RARE anatomical scan was collected for each rat with the following parameters: TR/TE = 8500/30 ms; matrix size = 120 × 120; field of view = 30 × 30 mm; and voxel size = 250 × 250 × 500 μm. Sixty axial slices were collected with a slice gap of 0 mm and an excitation angle of 90 degrees. Next, a high resolution spin echo 2D DTI scan was acquired for each rat with the following parameters: TR/TE = 3025/19 ms; four EPI segments; bandwidth of 300 kHz; and 30 non-collinear gradient directions with a single b-value shell at 1000 s/mm^2^ and five images with a b-value of 0 s/mm^2^ (referred to as B0). Comparable to the acquired T1 scans, sixty axial slices were collected for DTI with a slice gap of 0 mm; matrix size = 120 × 120; field of view = 30 × 30 mm; and voxel size = 250 × 250 × 500 μm. A single shot PRESS shimming protocol was applied to increase field homogeneity around the rodent brain and collect a baseline B0 map prior to scan acquisition. Total scan time did not exceed one hour per session for each animal. During scanning, rats were anesthetized under 1–3% isoflurane in oxygen, maintained a body temperature of 34–36 degrees Celsius on a heated water bed, and were monitored for respiratory rates between 40 and 60 beats/min. Representative scans are depicted in Fig. 2. DTI data are available via request to the authors contingent upon potential co-authorship agreement and the creation of a formal data sharing agreement plan.

### Processing and statistical analysis

Once collected, all DTI scans were converted to NIFTI files and uploaded into MedInria v.1.9.2 (2010 INRIA—Asclepios Research Team) for scan analysis. Two experimenters blind to animal condition outlined two distinct sub-regions of the corpus callosum for analysis: the interhemispheric sub-region that is predominantly composed of commissural fibers (ICC) and the cortically-projecting lateral sub-regions (PCC). PCC was further subdivided into the left and right PCC to account for any lateralization in the neuroimaging data. Regions of interest were hand drawn due to a lack of early and late adolescent rat MRI atlases and to examine alcohol-related neuropathology appropriately. Reliable scanning data was collected for 7–12 rats per sex/postnatal treatment/intervention group. For three regions (forebrain, ICC, and left/right PCC), the following data were extracted from the two sets of scans and analyzed using SPSS statistical software (IBM): volume, fractional anisotropy, and axial/radial diffusivity. Mixed repeated measures ANOVAs were employed to investigate alterations to the *rate* of change to weight and brain structure *within-animal across adolescence* while cross-sectional two and three-way ANOVAs were conducted at each time point to explore significant main effects of sex, treatment, and intervention on corpus callosum myelination on PD30 or 42 only. Regression-based mediation analysis was performed to investigate relationships between brain-body growth. Collectively, these analyses aid in the evaluation of alcohol- or intervention-specific alterations to the trajectory of myelination in the adolescent brain.

### Ethics approval and consent to participate

All rats were acquired, cared for, and methodology was in accordance with the guidelines provided by the University of Delaware’s Institutional Animal Care and Use Committee, the NIH Guide for the Care and Use of Laboratory Animals, and comply with the ARRIVE guidelines.

## Results

### AE during the BGS prevents normative weight gain during neonatal and adolescent development

**Figure 3:**
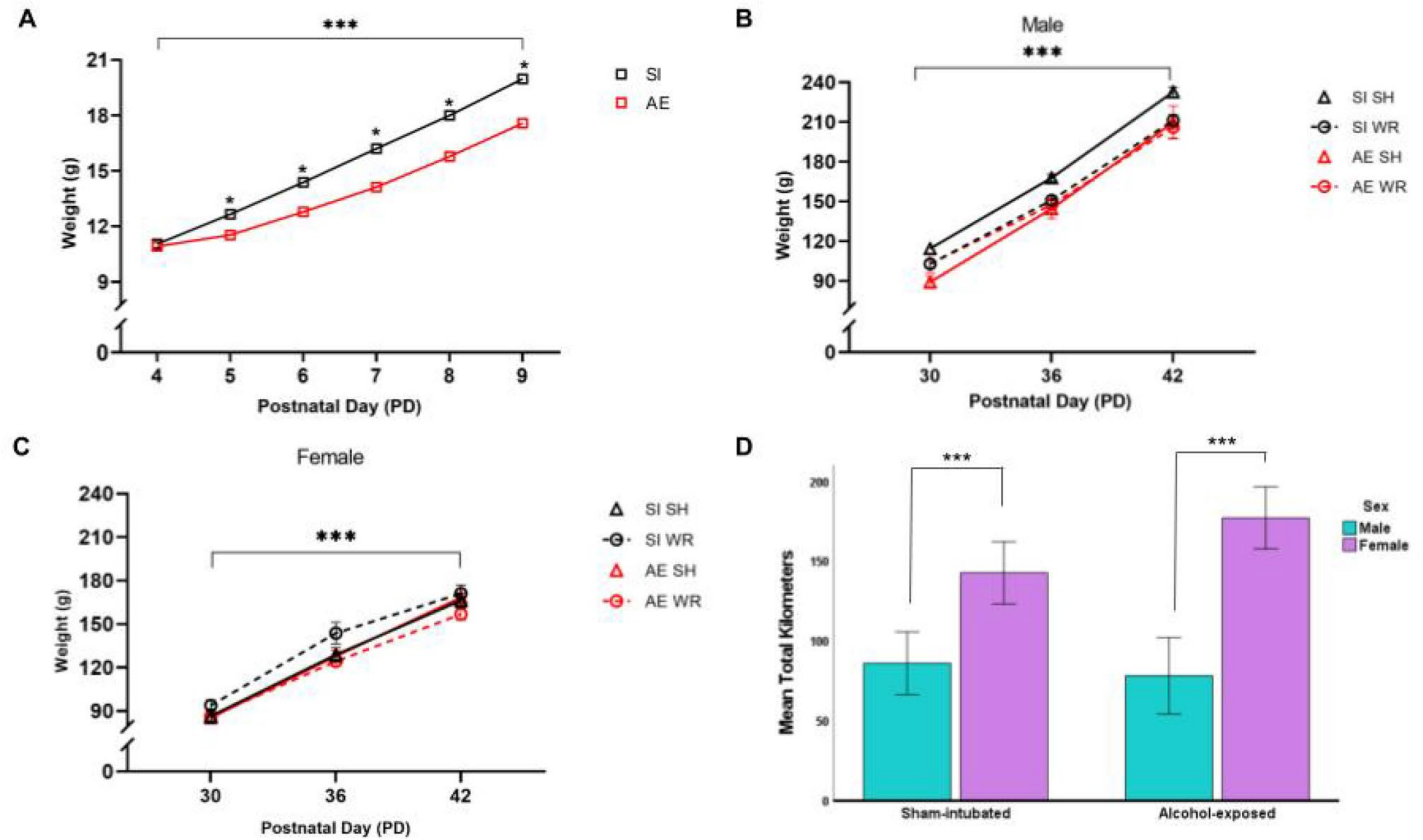
(**A**) Daily recorded mean body weight of AE (red) and SI (black) rat pups during the postnatal treatment period which targets the BGS. Mixed repeated measures ANOVA indicates that all rats gained weight across the treatment period (***p < .01). Cross-sectional analysis shows that AE pups weighed less than SI pups from PD five through nine during the treatment period (*p < .01). Data were collapsed across sex as there was no main effect or interaction of weight gain with sex. (**B**–**C**) Recorded mean body weight of male and female AE and SI rats from both intervention groups (WR and SH control) before, during, and after the intervention period. Mixed repeated measures ANOVAs indicate that all rats gained weight across the adolescent intervention period (***p < .001). There were significant main effects of intervention exposure and sex wherein intervention-exposed females gained the least amount of weight across the adolescent intervention period. Cross-sectional analysis shows that AE pups weighed less than SI rats at each time point during the intervention period. D) Average total kilometers run recorded from WR cages containing same-sex AE or SI rats. Female rats ran the most during the adolescent intervention period. There was no main effect of postnatal treatment group on distance run, suggesting that AE did not lead to significant motor deficits that impeded the capacity to run. n = 7–12 rats per sex/postnatal treatment/intervention group. Error bars are mean ± SEM.

Analysis using a mixed repeated measures ANOVA (within-subject variable: weight/day; between-subject variables: sex, postnatal treatment group; covariate: litter number) shows that rat pups from each postnatal treatment group gained weight during the treatment period (PD four through nine; Fig. 3A). However, there is a significant interaction between treatment day and treatment group (F_5, 380_ = 31.6, *p* < .001, η_p_^2^ = 0.30). Further investigation using two-way ANOVAs (sex × postnatal treatment group; covariate: litter number) confirm that AE pups weighed consistently less than SI control pups on PD five through nine (*p* < .001). The negative impact of AE on pups’ weight during the brain growth spurt was dampened by delivering an additional daily milk-only intubation to AE pups. There were no main or interactive effects of sex on weight gain during the postnatal treatment period. Additionally, we did not discover any effect of litter on neonatal weight gain despite culling and cross-fostering procedures. This is in line with other preclinical studies which have determined that such procedures have little to no impact on development or behavior^57–59^.

Analysis using a mixed repeated measures ANOVA (within-subject variable: weight/day; between-subject variables: postnatal treatment and intervention groups; covariate: sex) shows that juvenile rats from all treatment and intervention groups gained weight across adolescence (PD30, 36 and 42; Fig. 3B, C). We have found that on average female rats ran more than male rats during the intervention period (F_1, 17_ = 40.73, *p* < .001, η_p_^2^ = 0.71; see Fig. 3D). In line with this finding, there exist significant interactions between intervention day and sex or intervention group on weight gain, longitudinally (F_2, 148_ = 51.4, *p* < .001, η_p_^2^ = 0.41; F_2, 148_ = 4.5, *p* = .01, η_p_^2^ = 0.06, respectively). Collectively, these results show that female rats gained less weight across adolescence when compared to their male counterparts, as expected, and that access to a running wheel further prevented weight gain and disproportionately affected females, potentially due to the fact that female rats ran the most. While we did not directly measure caloric intake across the intervention period, it is well-established that male rats weigh more than their female counterparts during adolescence^60^.

Despite demonstrating that AE and SI control rats have similar rates of body growth across adolescence, two and three-way ANOVA analyses (postnatal treatment group × sex; postnatal treatment group × intervention group with sex as a covariate, respectively) conducted at each time point indicate that AE rats weighed consistently less than SI control rats on PD30, 36, and 42 (*p* < .001). To further probe these relationships, an analysis of the percentage of weight gained across adolescence was conducted using a three-way ANOVA (postnatal treatment group × intervention group with sex as a covariate). This analysis shows that there were main effects of sex (F_1,74_ = 17.8, *p* < .001, η_p_^2^ = 0.20) and intervention exposure (F_1,74_ = 6.7, *p* = .012, η_p_^2^ = 0.10) on the percentage of weight gain across adolescence wherein female WR rats had the smallest increase in weight gain between PD30 and 42. Finally, we find that pup weight on PD nine is positively correlated with weight on PD30 (r^2^ = 0.71) in both AE and SI control rats.Together, these findings indicate that AE during the BGS prevents age-appropriate weight gain in the neonatal period, ultimately affecting the growth trajectory of these rodents in adolescence. Finally, exercise intervention had no main or interactive effects on weight when analyzed cross-sectionally at each adolescent time point.

### AE during the BGS delays the trajectory of forebrain and corpus callosum growth in adolescence and is mediated by body weight

**Figure 4:**
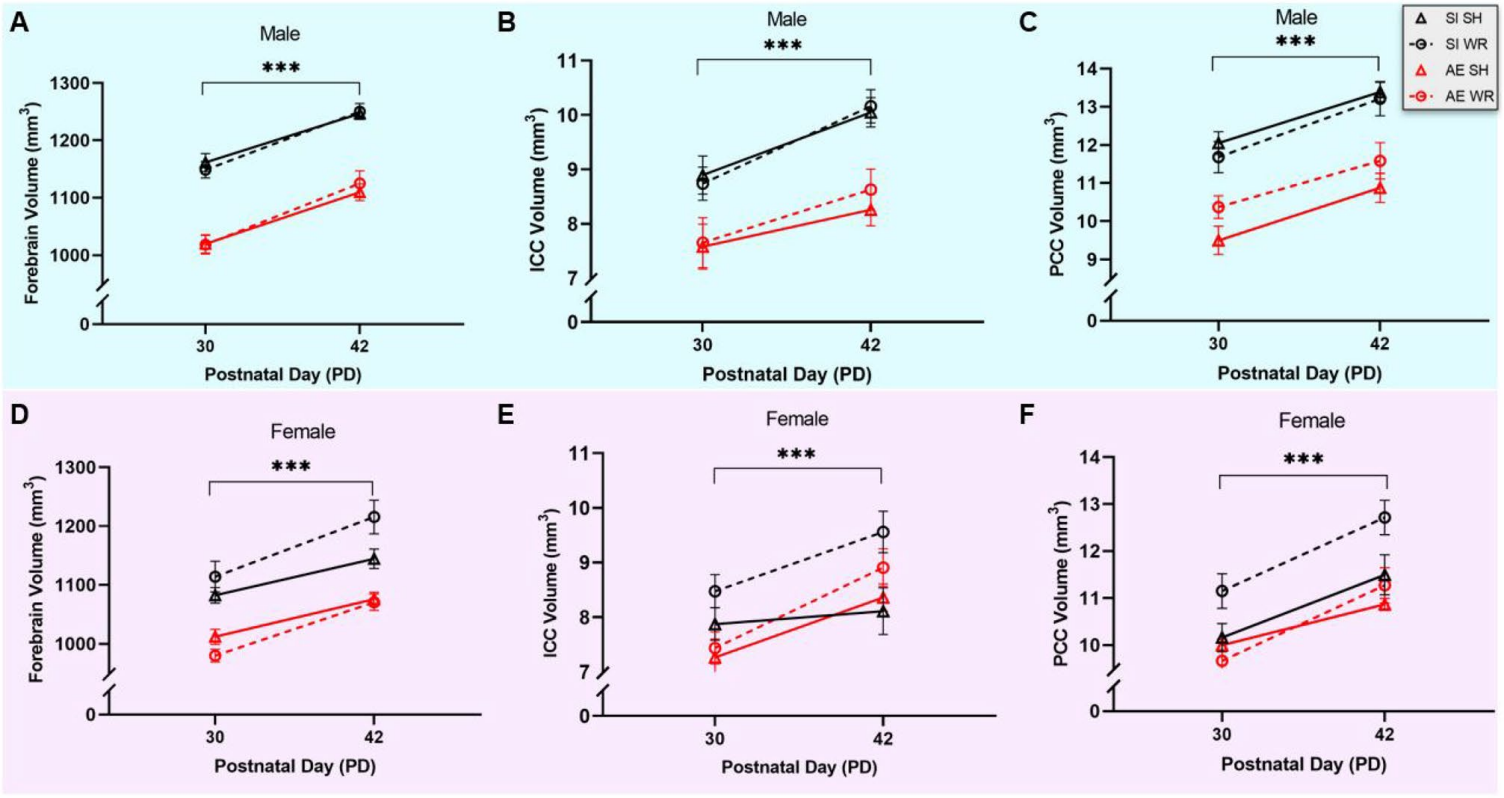
Average within-animal forebrain volume growth in male (**A**) and female (**D**) AE/SH, AE/WR, SI/SH, and SI/WR rats during the intervention period. Mixed repeated-measures ANOVA indicates that forebrain volume increases between PD30–42 in all rats (***p < .001) and there is a significant interaction with intervention exposure. Average within-animal ICC volume growth in male (**B**) and female (**E**) AE/SH, AE/WR, SI/SH, and SI/WR rats during the intervention period. Mixed repeated-measures ANOVA indicates that ICC volume increases between PD30–42 in all rats (***p < .001). Average within-animal PCC volume growth in male (**C**) and female (**F**) AE/SH, AE/WR, SI/SH, and SI/WR rats during the intervention period. Mixed repeated-measures ANOVA indicates that PCC volume increases between PD30–42 in all rats (***p < .001). n = 7–12 rats per sex/postnatal treatment/intervention group. Error bars are mean ± SEM.

To investigate the main and interactive effects of AE during the BGS and exercise intervention exposure on corpus callosum growth between juvenile and adolescence periods, mixed repeated measures ANOVAs were used (within-subjects variable: weight/day; between-subjects variables: postnatal treatment and intervention groups; covariate: sex). We found that forebrain volume increased in all juvenile rats in this study, as predicted (PD30–42; F_1, 75_ = 250.9, *p* < .001, η_p_^2^ = 0.77). Additionally, there is a significant interaction between age and intervention group (F_1, 75_ = 7.9, *p* = .006, η_p_^2^ = 0.10) indicating that exercising increases the rate of forebrain growth across adolescence in all rats that had free access to a running wheel. Similar analyses show that ICC and PCC volumes increased with age in all rats (F_1, 75_ = 29.0, *p* < .001, η_p_^2^ = 0.28; F_1, 75_ = 79.4, *p* < .001, η_p_^2^ = 0.51, respectively). Yet, there was no interaction of ICC or PCC growth with postnatal treatment exposure, exercise intervention, or sex. Collectively, these data show that the rate of forebrain, ICC and PCC growth across adolescence was unchanged by AE, but that the rate of forebrain growth was immediately increased by aerobic exercise intervention in all rats. All within-subjects analyses are depicted in the graphs in Fig. 4.

Further analysis using two and three-way ANOVAs (postnatal treatment × sex; postnatal treatment × intervention exposure with sex as a covariate, respectively) were conducted for data collected on PD30 or 42 to investigate group differences in forebrain, ICC, and PCC volumes at each time point. First, we discovered that there exists a main effect of postnatal treatment on the volumes of forebrain, ICC, and PCC when measured at either time point (*p* < .05), illustrating that AE during the BGS results in a lasting reduction to the volumes of the brain and corpus callosum subregions in adolescence. Importantly, the previously described longitudinal analysis confirms that AE does not perturb the rate of growth of these structures between the chosen time points. Second, we found a main effect of sex on forebrain, ICC, and PCC volumes when measured at both time points. This relationship shows that the female brain and its subregions are smaller than its male counterparts in adolescence, consistent with the literature^61–63^. Moreover, there is a significant interactive effect between sex and postnatal treatment group on ICC and PCC volumes which is depicted in the graphs in Fig. 4B and E (male comparison) and Fig. 4C and F (female comparison). These data indicate that AE during the BGS disproportionately affects the volumes of ICC and PCC in males compared to females across late development. Indeed, the male brain is more vulnerable to AE-related impairments to growth and maturation. Since the female ICC and PCC are smaller at baseline and AE does not change ICC or PCC volume in adolescence, the volumes of male AE CC are comparable to those in the AE and SI female brain on PD30 and 42.

Finally, we observed a positive effect of exercise intervention exposure on ICC volume in late adolescence for female rats. Our data show that wheel running exposure significantly increased the volume of ICC on PD42 in AE and control SI female rats only. However, given the fact that AE did not lead to reductions in ICC volume in the female adolescent brain, we cannot conclude that the observed intervention-related findings indicate a restoration of alcohol-induced damage to CC. Nevertheless, when considering the elevated distance run by female rats compared to males in this study, it can be predicted that increasing exercise exposure for male rats in future studies might mitigate the alcohol-induced reduction to ICC and PCC volume observed in the male adolescent brain. Cross-sectional results are summarized in Table 1.

**Table 1:** Cross-sectional analysis of forebrain, ICC, and PCC volume data collected on PD30 and 42.

	Forebrain volume		ICC volume		PCC volume	
	PD30	PD42	PD30	PD42	PD30	PD42
**Main effects**						
Sex	***F < M	n/a	***F < M	n/a	**F < M	n/a
PN treatment	***AE < SI	***AE < SI	***AE < SI	***AE < SI	***AE < SI	***AE < SI
Intervention	n/a	*n.s*	n/a	*SI < WR	n/a	*n.s*
**Covariates**						
Sex	n/a	***F < M	n/a	*F < M	n/a	*F < M
Interactions	*n.s*	*n.s*	*Sex × PN treatment	*Sex × intervention *Sex × PN treatment	*Sex × PN treatment	*Sex × PN treatment

Significant values are in bold.

***p < .001, **p < .01, *p < .05; *n/a* variable not included in analysis, *n.s.* non-significant difference.

**Figure 5:**
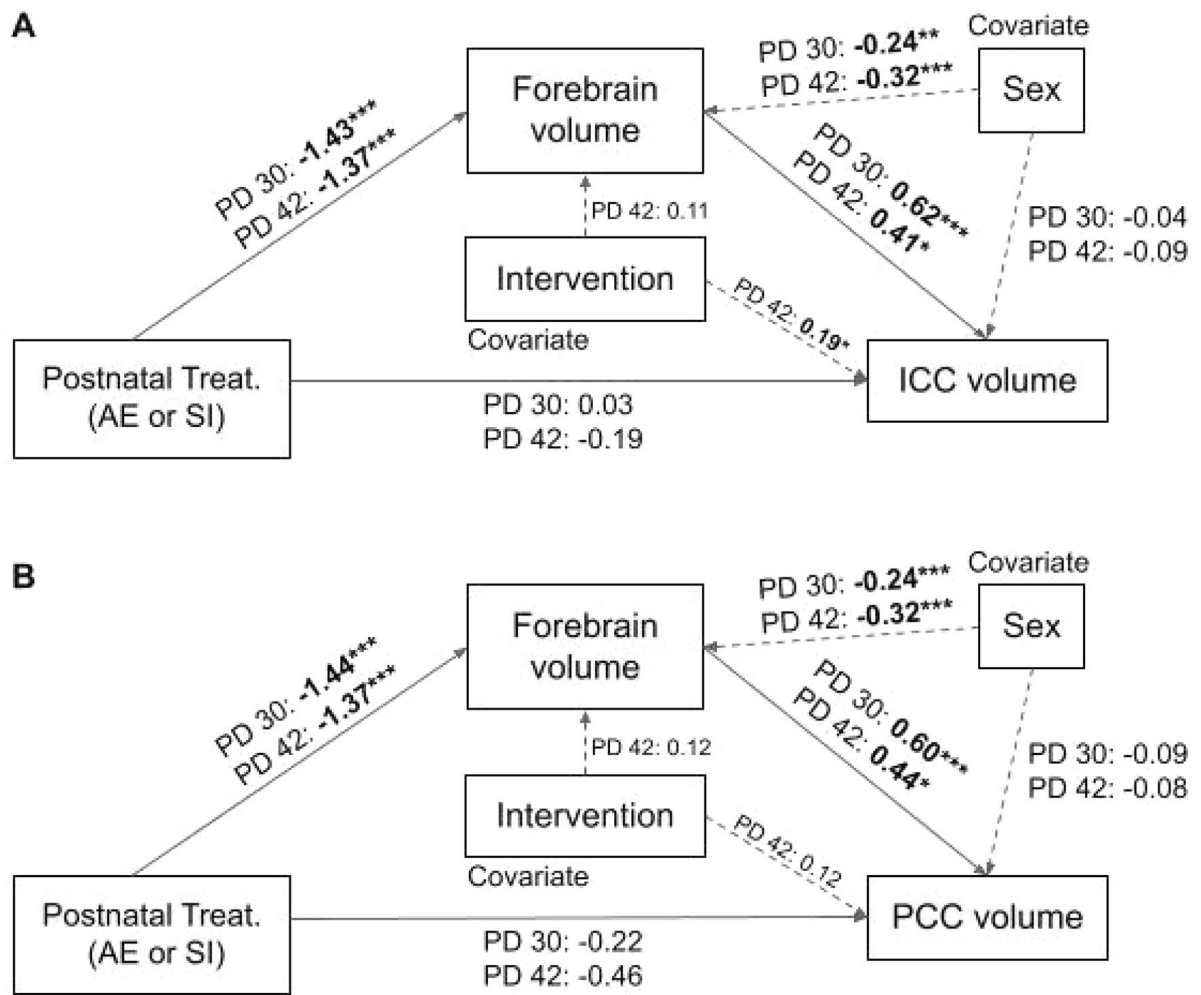
Statistical models for the regression-based mediation analyses which illustrate that postnatal treatment indirectly affects ICC (**A**) and PCC (**B**) volumes through lasting alterations to forebrain volume on PD30 and 42. All values represent standardized regression coefficients. Bolded coefficients indicate statistically significant relationships. Factors: postnatal treatment, ICC/PCC volume; Mediator: Forebrain volume; Covariates: sex and intervention group. N = 80, ***p < .0001, **p < .01, *p < .05.

Regression-based mediation analysis with bootstrapping using the PROCESS v3.5 function for SPSS confirms that the impact of postnatal treatment on ICC (Fig. 5A) and PCC (Fig. 5B) volume is mediated by alterations to forebrain volume on PD30 and 42. When sex is included as a biological factor (covariate) in the analysis, it is evident that sex also has an effect on forebrain volume and, thus, indirectly affects ICC/PCC volume in adolescence.

**Figure 6:**
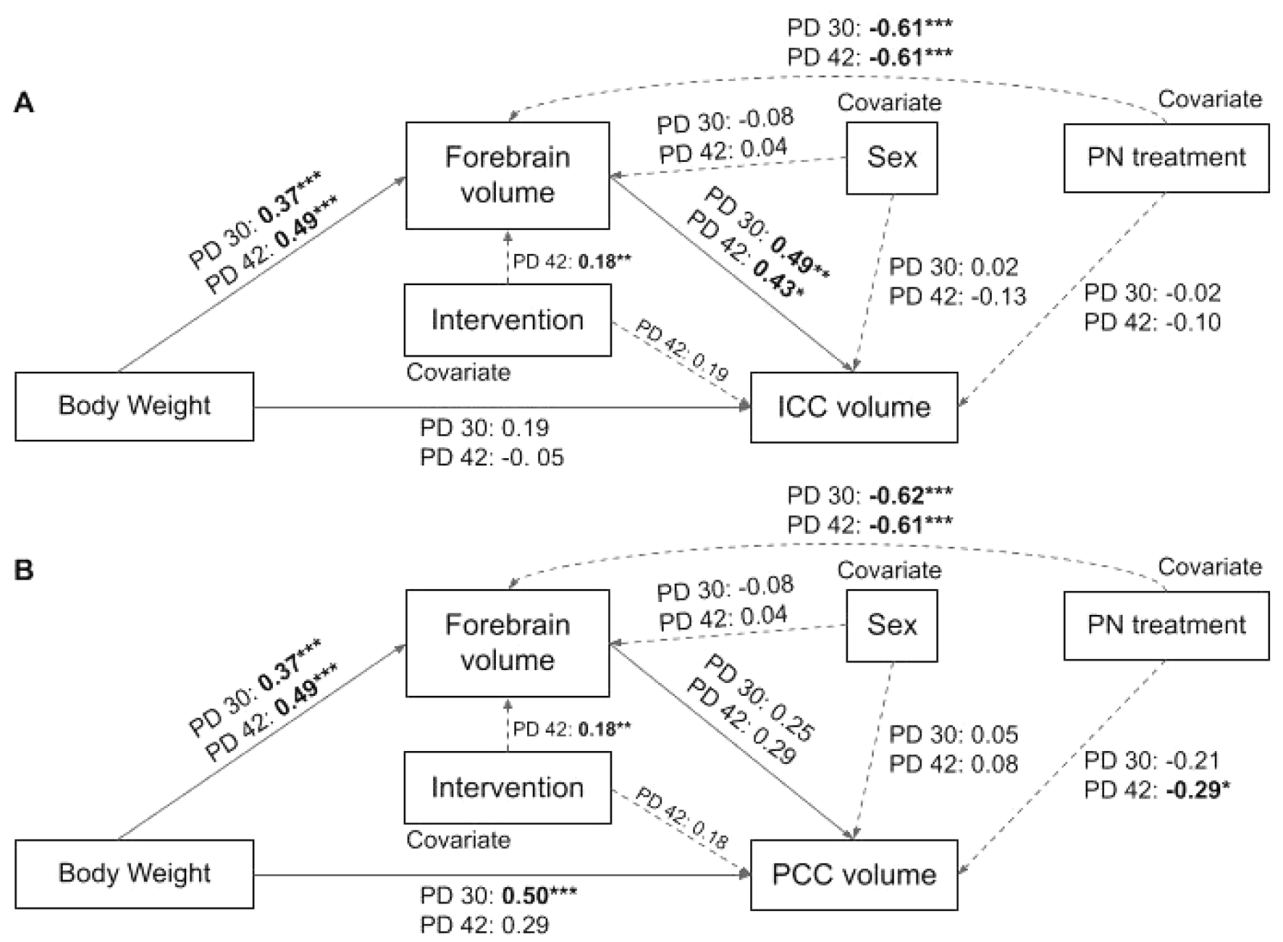
Statistical models for the regression-based mediation analyses which illustrate that body weight indirectly affects ICC (**A**) but not PCC (**B**) volumes through alterations to forebrain volume on PD30 and 42. Conversely, body weight influences PCC volume directly on PD30 only. All values represent standardized regression coefficients. Bolded coefficients indicate statistically significant relationships. Factors: body weight, ICC/PCC volume; Mediator: Forebrain volume; Covariates: sex, postnatal treatment group, and intervention group. N = 80, ***p < .0001, **p < .01, *p < .05.

Given the lasting effect of AE during the BGS on developmental weight gain, we then explored the potential relationship between body weight, forebrain volume, and ICC/PCC volume in adolescence. We show through regression-based mediation analysis that weight on PD30 and 42 has an indirect effect on ICC volume as mediated through total forebrain volume in both AE and SI control rats (Fig. 6A). However, this relationship is not maintained when comparing the mediation effect of forebrain volume on the body weight-PCC volume relationship. Instead, body weight directly affects PCC volume on PD30 only (Fig. 6B). Moreover, postnatal treatment group (covariate) influences weight and forebrain volume, indirectly impacting ICC/PCC volumes on PD30 and 42. Despite the main effects of sex and intervention exposure on the absolute and percent of weight gain across adolescent, sex had no effect on forebrain or ICC/PCC volumes in our model. Intervention exposure had an observed effect on forebrain volume only in this model. Mediation results are further described in Table 2. Together, these analyses suggest that adolescent weight gain may contribute to whole and regional brain growth and that postnatal treatment has a significant impact on this mediating relationship. The results described in Sects. 3.1 and 3.2 illustrate that sexual dimorphisms in the rate of or percent increase in body weight that occurs in adolescence, likely due to pubertal onset^64^, do not affect the observed relationship between weight and forebrain/CC volume. One limitation of this analysis is that it does not comprehensively account for sex or postnatal-treatment specific alterations to the independent variable (body weight).

**Table 2:** Regression-based mediation analyses illustrating the indirect effects of forebrain volume on ICC/PCC volume for PD30 and 42.

Indirect path	Time point	Estimated effect	95% CI		Ratio to total effect
			LL	UL	
PN treatment → forebrain volume → ICC volume	PD 30	− 0.90	− 1.4	− 0.53	87%
PN treatment → forebrain volume → ICC volume	PD 42	− 0.56	− 1.02	− 0.07	59%
PN treatment → forebrain volume → PCC volume	PD 30	− 0.86	− 1.33	− 0.43	57%
PN treatment → forebrain volume → PCC volume	PD 42	− 0.61	− 1.05	− 0.21	38%
Body weight → forebrain volume → ICC volume	PD 30	0.18	0.06	0.37	37%
Body weight → forebrain volume → ICC volume	PD 42	0.01	0.0004	0.0164	> 99%
Body weight → forebrain volume → PCC volume	PD 30	0.0059	− 0.0005	0.0135	n/a
Body weight → forebrain volume → PCC volume	PD 42	0.0043	− 0.0009	0.0105	n/a

If a 0 falls within the bounds of the bootstrapping confidence interval (CI), then the proposed relationship does not exist.

N = 80, bootstrapping sample size = 500, *LL* lower limit, *UL* upper limit, *CI* confidence interval, all effects are partially standardized.

Furthermore, since we have established that weight on PD nine is positively correlated with weight on PD30 in all rats, it can also be concluded that acute reductions in weight gain resulting from AE during the BGS, prevents age-appropriate weight gain in adolescence, and ultimately contributes to delayed forebrain and white matter tract growth in adolescence irrespective of sex. It should be noted that while exercise intervention reduced weight gain across adolescence, it did not cause weight loss in our paradigm. Importantly, forebrain volume was increased by exercise intervention exposure. This presents one caveat to the observed mediation effect, likely due to the therapeutic effect that aerobic exercise has on myelinogenesis, and thus brain volume, during juvenile neurodevelopment. Thus, postnatal treatment (AE) and intervention exposure (WR) are shown to have opposite effects on forebrain volume, suggesting a potential restorative effect of adolescent exercise intervention in our rodent model of FASD.

### AE during the BGS delays the myelination of multiple sub-regions of corpus callosum in adolescence

As aforementioned, comparison of fractional anisotropy, axial diffusivity, and radial diffusivity data from DTI scans reveals alterations to brain microstructure. This study harnessed the power of noninvasive neuroimaging to assess the therapeutic potential of an adolescent exercise intervention to restore alcohol-induced changes to fractional anisotropy, axial diffusivity, and radial diffusivity in CC using a well-established rodent model of FASD. Of note, a paired samples t-test of data collected from left and right PCC indicates that there was a significant difference in DTI data obtained from left and right hemispheres across adolescence, an expected asymmetry that was not identified when evaluating volume data^65^.

**Figure 7:**
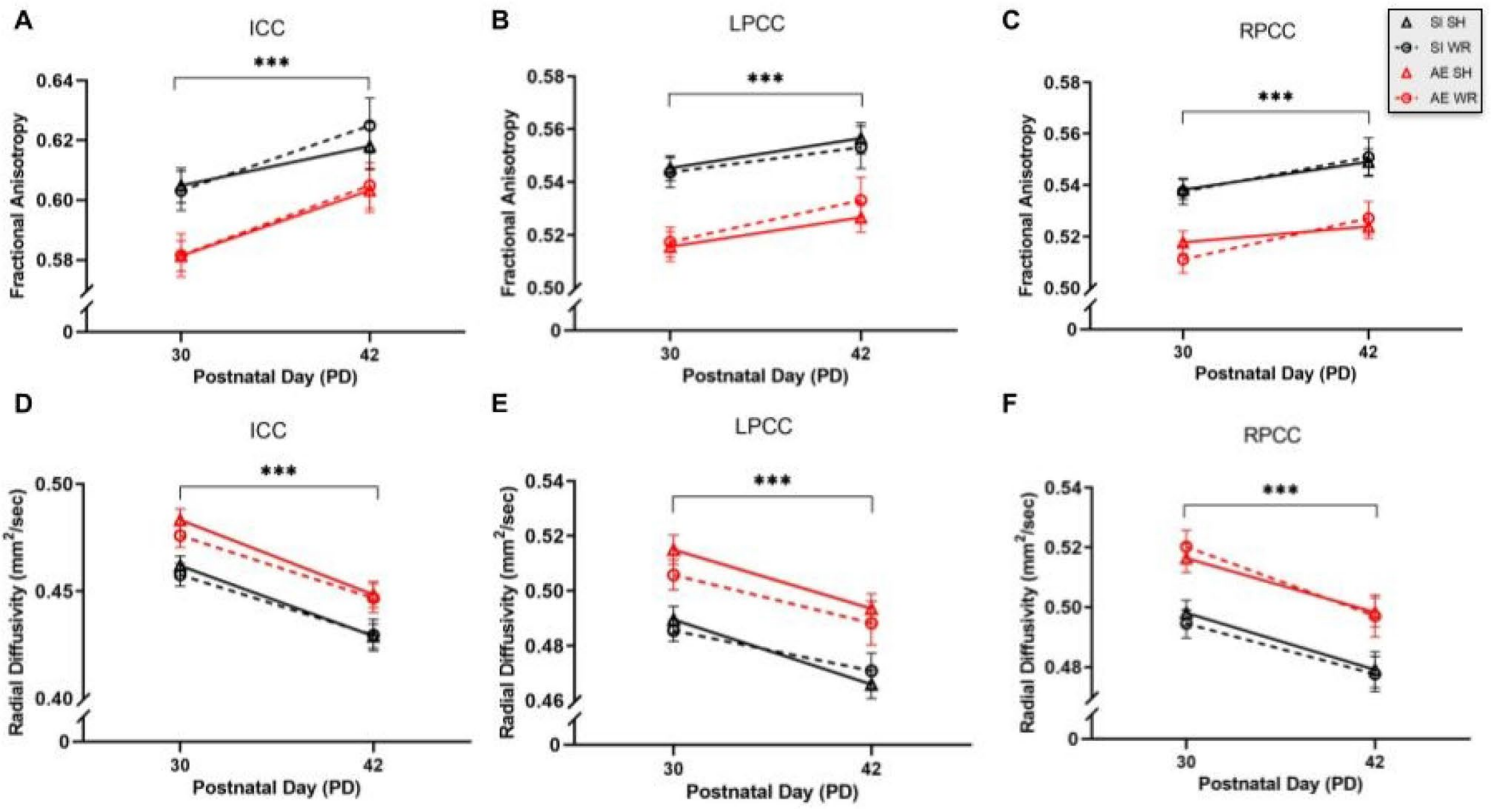
Average within-animal ICC, LPCC, and RPCC fractional anisotropy (**A**–**C**, respectively) and radial diffusivity (**D**–**F**, respectively) in AE/SH, AE/WR, SI/SH, and SI/WR rats during the intervention period. Mixed repeated measures ANOVA indicates that age-related alterations to fractional anisotropy and radial diffusivity are inversely proportional in all CC sub-regions (***p < .001). All data are shown collapsed across sex as the analyses indicate no main or interactive effects of sex on the DTI measures. n = 7–12 per sex/postnatal treatment/intervention group. Error bars are mean ± SEM.

Analysis using mixed repeated measures ANOVAs (within-subject variable: weight/day; between-subject variables: postnatal treatment and intervention groups; covariate: sex) indicate that fractional anisotropy increases in ICC and left/right PCC across development, as expected (Fig. 7A–C). We suspect that this is due to de novo axon myelination and/or thickening of existing myelin sheaths which occur naturally during adolescence for circuit refinement^66,67^. However, right PCC had lower fractional anisotropy values compared to left PCC on PD30 and 42 in all rats, perhaps due to a difference in the trajectory of maturation of myelin between brain hemispheres. Further, analysis with two and three-way ANOVAs (postnatal treatment × sex; postnatal treatment × intervention exposure with sex as a covariate, respectively) for data collected on PD30 and 42 show that fractional anisotropy values are lower in AE rats compared to SI control rats at both time points, indicating that water is moving more freely within CC in AE adolescent rats (Table 3).

**Table 3:** Cross-sectional analysis of ICC and left/right PCC fractional anisotropy and radial diffusivity data collected on PD30 and 42.

	ICC fractional anisotropy		ICC radial diffusivity	
	PD30	PD42	PD30	PD42
**Main effects**				
Sex	*n.s*	n/a	*n.s*	n/a
PN treatment	***AE < SI	*AE < SI	***SI < AE	***SI < AE
Intervention	n/a	*n.s*	n/a	*n.s*
**Covariates**				
Sex	n/a	*n.s*	n/a	*n.s*
Interactions	*n.s*	*n.s*	*n.s*	*n.s*

	LPCC fractional anisotropy		LPCC radial diffusivity	
	PD30	PD42	PD30	PD42
**Main effects**				
Sex	*n.s*	n/a	*n.s*	n/a
PN treatment	***AE < SI	***AE < SI	***SI < AE	***SI < AE
Intervention	n/a	*n.s*	n/a	*n.s*
**Covariates**				
Sex	n/a	*n.s*	n/a	*n.s*
Interactions	*n.s*	*n.s*	*n.s*	*n.s*

	RPCC fractional anisotropy		RPCC radial diffusivity	
	PD30	PD42	PD30	PD42
**Main effects**				
Sex	*n.s*	n/a	*n.s*	n/a
PN treatment	***AE < SI	***AE < SI	***SI < AE	**SI < AE
Intervention	n/a	*n.s*	n/a	*n.s*
**Covariates**				
Sex	n/a	*n.s*	n/a	*n.s*
Interactions	*n.s*	*n.s*	*n.s*	*n.s*

Significant values are in bold.

***p < .001, **p < .01, *p < .05; *n/a* variable not included in analysis, *n.s.* non-significant difference.

To explore the potential biomolecular mechanism underlying the observed AE-induced changes to CC integrity, we examined group differences in axial and radial diffusivity within ICC and PCC. Analysis using mixed repeated measures ANOVAs (within-subject variable: weight/day; between-subject variables: postnatal treatment and intervention groups; covariate: sex) indicates that radial diffusivity decreased in ICC and left/right PCC across adolescence as expected, suggesting that myelination increased in all animals across adolescence (Fig. 7D–F). However, analyses using two and three-way ANOVAs (postnatal treatment × sex; postnatal treatment × intervention exposure with sex as a covariate, respectively) for data collected on PD30 or 42 show that radial diffusivity values remained higher in AE compared to SI control rats in all corpus callosum subregions (Table 3). Moreover, there were no significant main effects of sex, postnatal treatment group, or intervention group on axial diffusivity values in ICC or left/right PCC across adolescence, confirming that the observed structural alterations did not result from changes to axonal integrity. These data are indicative of either a delay in the trajectory of corpus callosum myelination or of a reduction in the amount of myelination produced in the FASD-affected corpus callosum. Additionally, the observed alterations were not immediately mitigated by adolescent exercise intervention and were not sexually dimorphic. It should be noted that radial diffusivity values were negatively correlated with PCC volume on PD30 (r = − 0.45, *p* < .001) and 42 (r = − 0.37, *p* < .001) and with ICC volume on PD30 only (r = − 0.42, *p* < .001) in all rats. These relationships suggest that alcohol-induced changes to CC subregion volume (mediated by growth retardation) are correlated with changes to CC myelination across adolescence. Perhaps the inverse relationships indicate that CC subregion volumes are smaller when there exists less myelination (increasing radial diffusivity values).

## Discussion

### Summary of findings

This study employed a rodent model of FASD to explore the potential beneficial effect of an aerobic exercise intervention on mitigating alcohol-induced pathology to corpus callosum development and myelination in the adolescent brain. Concordant to findings described in the clinical literature, AE during the BGS was shown to alter age-appropriate weight gain across neonatal and adolescent development^68^. Regression-based mediation analysis shows that the effect of postnatal treatment on body weight indirectly affects corpus callosum growth (volume) through alterations to forebrain volume in adolescence. Importantly, aerobic exercise intervention (voluntary wheel running) in adolescence had the opposite effect on forebrain volume, stimulating forebrain growth in all rats. Additionally, we were the first to show that alcohol-induced alterations to several of these measures across adolescence were discovered to be sexually dimorphic—body weight, forebrain volume, and corpus callosum (ICC and PCC sub-regions) volume. ICC and PCC volumes were largest in SI control female rats exposed to the adolescent exercise intervention. Females were observed to run greater distances on average when exposed to the adolescent intervention compared to their male counterparts which likely contributed to sexual dimorphisms in weight gain and forebrain growth across adolescence. A follow-up study wherein caloric intake is measured during intervention exposure is needed to confirm this remaining hypothesis.

Analysis of data collected from diffusion tensor imaging scanning demonstrates that CC myelination in adolescence is reduced by AE during the BGS. Due to the anatomical differences between commissural and projecting CC fibers, two sub-regions of CC—ICC and PCC—were examined. Longitudinal analysis confirmed that radial diffusivity values (an indirect measure of myelination) decreased in ICC and PCC between PD30 and 42 in all rats, as expected during this fundamental period of circuit refinement. However, cross-sectional analysis of DTI data collected on PD30 or 42 shows that radial diffusivity values remain higher in the AE ICC and PCC compared to controls, indicative of disrupted or hypomyelination. Adolescent exercise intervention had no immediate effect on radial diffusivity values in adolescence. Future research is needed to investigate if AE leads to a persistent loss of myelination in CC in adulthood or if the trajectory of this neurodevelopmental process is simply delayed and/or lengthened in cases of FASD.

Further, it was discovered that the progression of PCC myelination was incongruous in the left and right hemispheres such that myelination was greater in the right PCC in all rats. To our knowledge, this is the first examination of PCC myelination lateralization in rodents. However, much like in the human brain, hemispheric lateralization in the rat brain is a result of genetic predisposition and putative function/early-life experience. Structural and functional brain asymmetries play a key role in individual variations in motor capacity, emotional processing, and language ability in both the rodent and human brain^28^. For example, cortical lateralization is well-described and attributes to paw preference in rodents^69–71^. We did not find that lateralized PCC development resulted from AE during the BGS (potentially considered an early-life experience in our model of FASD). However, gestational alcohol exposure has been shown to yield limb preference in rodent models of FAS/FASD^72^.

### Body weight, brain size and white matter volume

As previously described, disruptions to normative growth trajectory in childhood have deleterious effects on brain development. Alcohol teratogenesis is a known risk factor for reaching age-appropriate growth and neurodevelopmental milestones^73,74^. Moreover, our data link FASD-related changes to body growth with impaired growth of salient white matter tracts in adolescent males. This is demonstrated by a reduction in ICC and PCC volumes in AE male rats on PD30 and 42. Furthermore, the AE CC in males was comparable in volume to the volume of CC in both AE and SI female rats. While reductions to CC subregion volume were mediated by overall reductions to forebrain volume, DTI scanning provided additional evidence that elucidated hypomyelination as another factor contributing to volumetric anomaly. Indeed, PCC volume at each time point was negatively correlated with radial diffusivity values such that the smaller the subregion, the more water was able to move around in the structure. A similar relationship was observed on PD30 in ICC. These relationships suggest that reductions to CC volume could be attributed, at least in part, to a decrease in tract myelination. Since the smallest CC were observed in adolescent male rats with previous AE, it is possible to connect this structural deficit with exposure to alcohol during development. Moreover, it is known that the male brain has a higher susceptibility to structural deficits resulting from alcohol teratogenesis^63,75^. Further exploration of this sex-specific vulnerability would benefit from histological analysis of brain tissue from a preclinical model of FASD wherein measures of myelination (i.e. myelin basic protein concentration, oligodendrocyte number, thickness of the myelin sheath) could be quantified.

Finally, we discovered a therapeutic effect of aerobic exercise intervention in adolescence on the rate of forebrain growth in AE and control rats. This relationship exists as a caveat to the observed body weight-forebrain volume relationship wherein exercise-related reductions to weight gain across adolescence were negatively correlated with rate of forebrain growth. Wheel running promoted brain growth in AE and SI rats of both sexes, as expected. Furthermore, it was observed that the volume of the ICC in the female brain (AE and SI control) was significantly increased following intervention exposure. This effect was not observed in the male brain, likely due to the longer distances voluntarily run by female rodents in this study. Several studies indicate that when provided with free access to running wheels, female rodents run more on average than males^76^. It could be predicted that intervention exposure would have the largest effect on changes to myelination in the female brain.

### Understanding the observed AE-related changes to CC structure: does alcohol exposure during the BGS perturb the trajectory of corpus callosum myelination or reduce the amount of myelination produced in the adolescent brain altogether?

The mechanism by which prenatal AE reduces CC myelination and causes neuroanatomical reorganization across the lifespan is unknown. Identification of these neurobiological processes is critical for evaluating the immediate and long-term effectiveness of behavioral interventions on mitigating impairments to myelination in cases of FASD. Several barriers impede our investigation into the teratogenic impact of AE on white matter tract development in humans at the cellular level. First, brain myelination is a postnatal process that continues over several decades in humans. Longitudinal studies spanning over 30 years have a lower feasibility, and results may be difficult to interpret given that many life experiences encountered during the first 30–40 years of life (i.e. socioeconomic status, malnutrition) may significantly affect the trajectory of myelination in the brain. Several clinical studies assessing the impact of AE during the second to third trimesters on CC volume and fractional anisotropy values through infancy, childhood, and adolescence describe unique changes to white matter structure across development. Briefly, noninvasive neuroimaging studies demonstrate that changes to CC volume and fractional anisotropy do not appear until at least two years of age in FASD-affected children^77^. During early childhood, from 2 to 7 years of age, FASD-affected children exhibit a reduction in CC volume with an increase in CC fractional anisotropy, demonstrating a precocial myelination of white matter^78^ in early-life in response to alcohol teratogenesis^30^. However, in late childhood/early adolescence, between 7 and 15 years of age, history of prenatal AE has the opposite effect on fractional anisotropy in CC, indicating hypomyelination. CC remains reduced in volume compared to typically-developing controls, and fractional anisotropy values are reduced in CC^45,79–81^. CC volume and fractional anisotropy remain reduced in the brains of FASD-diagnosed subjects throughout adolescence^63,82^ and adulthood^83^. More research is needed to understand the underlying anatomical and physiological mechanisms relating to measurable changes in anisotropy and diffusivity across development.

A review of the literature reveals two central hypotheses regarding the effect of prenatal AE on white matter tract development: (1) prenatal AE leads to precocial development of corpus callosum in childhood, and (2) prenatal AE leads to a delay in the trajectory of corpus callosum development in adolescence. It is unclear if these hypotheses are mutually exclusive. Our data support the latter hypothesis only as we did not scan during the neonatal period to confirm that myelination is delayed in early life. Indeed, values of radial diffusivity in AE CC on PD42 (late adolescence, analogous to neurodevelopment at about 22–25 years of age in humans) are comparable to those recorded in the control brain on PD30 (early adolescence, analogous to neurodevelopment at about 18 years of age in humans).

Preclinical research using animal models of FASD allow for the investigation of myelin-related neuropathology across the lifespan which validates noninvasive neuroimaging findings and is necessary for identifying underlying neurobiological mechanisms associated with alcohol-induced reductions to myelination. Further, such studies are ideal for exploring long-term deficits associated with FASD and for evaluating the effectiveness of behavioral interventions on circuit refinement in the adolescent brain. Myelination is supported by mature and precursor oligoglia which are largely proliferating during the second and third trimesters of human gestation when they are also most vulnerable to alcohol-induced apoptosis^84^. Therefore, resulting CC neuropathologies vary with the timing and amount of prenatal AE, creating an obstacle for systematic clinical investigation. For example, severe prenatal AE resulting in Fetal Alcohol Syndrome has been shown to lead to full or partial CC agenesis in school-aged children and adolescents^85^ while studies examining FASD-affected adolescents report a more moderate effect of prenatal AE on white matter development in CC^27^.

Several factors contribute to hypomyelination of CC. These include but are not limited to a reduction in the number of OPCs (depletion of the progenitor pool) through apoptosis or disrupted genesis during early development, dysregulated differentiation of OPCs to myelinating oligodendrocytes, apoptosis of myelinating oligodendrocytes, a thinning of the myelin sheath, and a reduction to the number of axons ensheathed by oligodendrocyte processes. Teratogenic AE leads to immediate apoptosis of mature oligodendrocytes and shifts the ratio of the total number of OPCs to the total number of mature oligodendrocytes in favor of precursor cells in the neonatal brain. OPC differentiation is initiated by an epigenetic modification which alters nucleosomal histone deacetylation during the BGS in rodent CC^86^. Further, it is postulated that mature oligoapoptosis is mediated by extracellular and internal signaling pathways^30^. Collectively, it is evident that oligoglia are most vulnerable to the teratogenic effects of alcohol during the pre-myelinating stage when myelin basic protein is being produced and oligoglia processes begin to flatten prior to ensheathment^84^. An immediate reduction to the number of OPCs and disruption of OPC differentiation depletes the OPC pool, leading to a sustained reduction in OPC population in adolescence and sometimes adulthood that contributes to CC hypomyelination later in life. Finally, prenatal AE results in neuroimmune system dysfunction^87,88^. Microglial and astrocytic proliferation and activity play a significant role in coordinating oligoglia proliferation, differentiation, and signaling to facilitate myelination during later stages of development^9,89,90^. Disruption of these pertinent glial interactions could contribute to the observed deficits in white matter tract development.

### Further evaluation of the effects of the proposed adolescent exercise intervention on white matter development in corpus callosum is needed

This study is the first step in a larger investigation into the potential therapeutic effect of an accessible and affordable behavioral intervention on stimulating myelination in the FASD adolescent brain. Further, we show that sexual dimorphisms exist in myelin pathology as a result of alcohol teratogenicity. Our results indicate an enhancing effect of aerobic exercise on forebrain growth and ICC volume growth, particularly in the female brain. However, it is possible that wheel running would have a similar effect on the male brain if male rats ran a comparable distance during the intervention period. At present, our findings do not indicate an immediate effect of aerobic exercise intervention on myelination in the adolescent brain of AE and SI rats. However, other immediate benefits of increased aerobic exercise on myelination may be identifiable at the molecular or ultrastructural levels. Given the propensity for aerobic exercise to stimulate neuroplasticity, it is expected that alterations to myelin plasticity will be observed at some level. Indeed, it is known that aerobic exercise upregulates OPC proliferation and differentiation as well as thickens the existing myelin sheath^14^. We predict that exercise intervention might upregulate the proliferation of OPCs in CC in adolescence to counteract any severe reductions to the OPC pool that may contribute to the hypomyelination observed in the AE brain. Additionally, existing sheaths are expected to be thickened following aerobic exercise intervention.

### Limitations and future research

This study is the first to use noninvasive longitudinal neuroimaging to examine the therapeutic effect of an adolescent exercise intervention on CC myelination in a rodent model of FASD. Our data indicate that increased aerobic exercise via voluntary wheel running did not immediately alter CC myelination when observed using DTI measures. Future studies should continue to collect DTI scans at later stages of adolescence and into adulthood to verify that exercise has no impact on CC myelination and to better conceptualize how the observed alcohol-related deficits to CC myelination contribute to the trajectory of brain circuit refinement in adolescence. Moreover, interpretation of these data would be enhanced by the analysis of simultaneously acquired magnetic transfer scans^91^.

Targeted and early intervention strategies are essential for preventing secondary disabilities and the emergence of maladaptive behaviors in adults diagnosed with FASD. Given the rising prevalence of FASD in the US coupled with the difficulties regarding correct FASD diagnosis^23^ the application of effective behavioral interventions is appealing to clinical professionals and caregivers. Results from future studies should elucidate: (1) the most effective timing for intervention onset, (2) the shortest intervention period with the greatest benefit for supporting neurodevelopment, and (3) further explore any sexual dimorphisms in intervention effectiveness to additionally tailor therapeutic development for the individual.

## Conclusions

Prenatal alcohol exposure resulting in a Fetal Alcohol Spectrum Disorders (FASD)-related diagnosis affects 1 in 20 live births in the United States annually, presenting a growing public health crisis that is greater than the risk for developing other significant neurodevelopmental disorders like Autism Spectrum Disorder or ADHD^92,93^ FASD-affected adolescents present with numerous social, cognitive and behavioral deficits associated with executive function that perturb perceptual reasoning, future planning, abstract conceptualization, learning, and memory^73^. Few therapeutic interventions exist for adolescents with FASD. However, preclinical studies using rodent models of FASD have explored potential pharmaceutical and behavioral interventions that may support late neurodevelopment and cognitive capacity in adulthood. In the study presented, we used non- invasive neuroimaging of a rodent model of FASD for optimal translatability via a longitudinal analysis. Notably, our findings support the theory that FASD delays the trajectory of corpus callosum myelination in adolescence. Further, they suggest that voluntary aerobic exercise in adolescence supports forebrain and corpus callosum growth, particularly benefitting the maturation of commissural fibers that are imperative for abstract and perceptual reasoning. While we did not uncover any immediate impacts of exercise intervention on restoring specific impairments to CC myelination, it remains unknown how this intervention alters acute myelination at the cellular level and/or if voluntary exercise yields any long-term benefits to CC myelination that may enhance adult cognition. In summary, innovative intervention programs which include adolescent interventions like the FAST club^94^ and LEAP program^95^ could prove most advantageous for resolving some of the underlying neuropathologies associated with FASD in adolescents and mitigate the resulting cognitive and behavioral deficits. While our DTI findings did not make apparent the mechanism behind the change in CC growth after aerobic exercise, CC volume changes were evident and this study contributes substantial evidence that exercise may be a positive effector not only for neuroplasticity, but also for myelin plasticity in a model of FASD.

## Data Availability

DTI data are available via request to the authors (PI, Dr. Anna Klintsova or first author, Katrina Milbocker) contingent upon potential co-authorship agreement and the creation of a formal data sharing agreement plan.

## Abbreviations

CC: Corpus callosum
OPC: Oligodendrocyte precursor cell
BGS: Brain growth spurt
MBP: Myelin basic protein
FASD: Fetal Alcohol Spectrum Disorders
AE: Alcohol exposure
DTI: Diffusion tensor imaging
PD: Postnatal day
SI: Sham-intubated
BAC: Blood alcohol concentration
WR: Wheel running
SH: Socially-housed control
ICC: Interhemispheric subregion of the corpus callosum
PCC: Cortically-projecting lateral subregions of the corpus callosum
